# A library of quantitative markers of seizure severity

**DOI:** 10.1111/epi.17525

**Published:** 2023-02-17

**Authors:** Sarah J. Gascoigne, Leonard Waldmann, Gabrielle M. Schroeder, Mariella Panagiotopoulou, Jess Blickwedel, Fahmida Chowdhury, Alison Cronie, Beate Diehl, John S. Duncan, Jennifer Falconer, Ryan Faulder, Yu Guan, Veronica Leach, Shona Livingstone, Christoforos Papasavvas, Rhys H. Thomas, Kevin Wilson, Peter N. Taylor, Yujiang Wang

**Affiliations:** ^1^ Computational Neurology, Neuroscience & Psychiatry Lab, Interdisciplinary Computing and Complex BioSystems Group, School of Computing Newcastle University Newcastle Upon Tyne UK; ^2^ Technical University Munich Munich Germany; ^3^ UCL Queen Square Institute of Neurology London UK; ^4^ NHS Greater Glasgow and Clyde Glasgow UK; ^5^ Department of Computer Science University of Warwick Warwick UK; ^6^ School of Mathematics, Statistics, and Physics Newcastle University Newcastle Upon Tyne UK; ^7^ Faculty of Medical Sciences Newcastle University Newcastle Upon Tyne UK

**Keywords:** computational neurophysiology, electroencephalography (EEG), seizure severity

## Abstract

**Objective:**

Understanding fluctuations in seizure severity within individuals is important for determining treatment outcomes and responses to therapy, as well as assessing novel treatments for epilepsy. Current methods for grading seizure severity rely on qualitative interpretations from patients and clinicians. Quantitative measures of seizure severity would complement existing approaches to electroencephalographic (EEG) monitoring, outcome monitoring, and seizure prediction. Therefore, we developed a library of quantitative EEG markers that assess the spread and intensity of abnormal electrical activity during and after seizures.

**Methods:**

We analyzed intracranial EEG (iEEG) recordings of 1009 seizures from 63 patients. For each seizure, we computed 16 markers of seizure severity that capture the signal magnitude, spread, duration, and postictal suppression of seizures.

**Results:**

Quantitative EEG markers of seizure severity distinguished focal versus subclinical seizures across patients. In individual patients, 53% had a moderate to large difference (rank sum r>.3, p<.05) between focal and subclinical seizures in three or more markers. Circadian and longer term changes in severity were found for the majority of patients.

**Significance:**

We demonstrate the feasibility of using quantitative iEEG markers to measure seizure severity. Our quantitative markers distinguish between seizure types and are therefore sensitive to established qualitative differences in seizure severity. Our results also suggest that seizure severity is modulated over different timescales. We envisage that our proposed seizure severity library will be expanded and updated in collaboration with the epilepsy research community to include more measures and modalities.

**Key Points**

- Existing measures of seizure severity can be complemented by objective quantitative markers of seizure EEG severity
- EEG‐based markers of seizure severity can distinguish clinically distinct seizure types
- Quantitative severity markers can be used to investigate fluctuations in seizure severity over time in individual patients

## 1 INTRODUCTION

Seizure severity is an important clinical measure for patients with epilepsy that is strongly correlated with quality of life.^1^ However, the best approach for measuring seizure severity remains unclear. Existing scales for measuring seizure severity, including the National Hospital Seizure Severity Scale (NHS3),^2^, ^3^ the Liverpool Seizure Severity Scale,^4^ and the Seizure Severity Questionnaire,^5^ are composed of questions on various aspects of seizures including warnings, ictal and postictal phenomena, and resultant injuries. Most scales separate seizures by their clinical classification^6^ to reflect differences in severity across different seizure types.

A primary shortcoming of existing measures of seizure severity is their reliance on patient or carer recollection.^6^ For example, a patient's recollection of their seizure may be impaired as a result of the seizure itself.^7^, ^8^ It is hence challenging to assess changes in severity from seizure to seizure in an unbiased manner for the full range of a patient's seizures. Objective, quantitative tools for measuring severity of individual seizures are therefore needed to understand variations in seizures on different timescales.

Electroencephalography (EEG)‐based severity markers are a potential approach to quantifying seizure severity. Past studies have used EEG features such as ictal duration^9^ and spatial synchronization^10^ as proxies for seizure severity. The anatomical spread of seizure activity has also been suggested as a measure of seizure severity.^6^ It is yet to be determined how such measures compare and which to use for each individual patient.

Moreover, various seizure features, which are directly associated with severity, fluctuate over time. For example, focal seizures are more likely to generalize in sleep,^11^ particularly in temporal lobe epilepsy (TLE).^12^ The extent of postictal suppression also depends on the time of day of seizure occurrence.^13^, ^14^ Subclinical seizures (without clinical symptoms) also follow circadian patterns.^15^ Furthermore, it has been shown that "seizure spatiotemporal evolutions"^16^ and ictal onset dynamics^17^ differ within individuals on circadian or longer timescales. Therefore, monitoring fluctuations in seizure severity could lead to a better understanding of an individual's epilepsy.

To objectively quantify seizure severity, we provide an expandable library of interpretable EEG‐based markers of seizure severity. As a way of validation, we test whether seizure severity markers distinguish clinically distinct seizure types^18^ with known differences in severity. We further show that markers of seizure severity are patient‐specific. As a proof of principle, we further demonstrate fluctuations in severity over circadian or longer timescales.

## 2 MATERIALS AND METHODS

### 2.1 Patient selection and data acquisition

This retrospective study analyzed iEEG recordings of 1009 seizures across 63 patients undergoing presurgical evaluation for medically refractory epilepsy. Seizure types were labeled by clinical teams according to International League against Epilepsy (ILAE) classifications: 656 focal, of which 232 were focal aware, 176 were focal impaired awareness, and 248 were uncategorized; 323 subclinical; six focal to bilateral tonic–clonic (FTBTC). Within this work, seizures with focal onset, clear clinical correlates, and no propagation to the contralateral hemisphere were labeled as focal seizures. Section S2 provides more details on the patient cohort.

Data were collected from two epilepsy monitoring units (EMUs) in the UK: University College London Hospitals and Glasgow, with 49 and 14 patients, respectively. Anonymized intracranial EEG (iEEG) recordings were analyzed following approval of the Newcastle University Ethics Committee (reference number 17042/2021). Electrographic seizure start and termination were labeled by clinical teams. Ictal periods were extracted with 2 min of pre‐ and postictal activity.

### 2.2 iEEG preprocessing

We first downsampled all EEG to 256 Hz. Preictal noise was detected using an iterative noise detection algorithm and visual inspection; noisy channels were removed from all seizures (see Methods S3.1). The iEEG was rereferenced to a common average reference, notch filtered at 50 and 100 Hz (2‐Hz window) to remove line noise, and band‐pass filtered between .5 and 100 Hz (fourth order, zero phase shift Butterworth).

### 2.3 Seizure markers

The selection of markers was inspired by seizure detection literature (e.g., Alotaiby et al.,^19^ Guo et al.,^20^ Birjandtalab et al.^19^, ^20^, ^21^). To quantify different types of features, our library of objective seizure severity markers has three main branches:

- "Peak" markers to measure the peak level of activity that occurs during a seizure;
- "Spatial" markers to summarize spread of ictal activity across recording channels; and
- "Suppression" markers to evaluate postictal suppression.

Ictal duration was also included as an additional severity marker.^22^ Table S3.1 gives detailed mathematical definitions of all markers.

Common notation is used throughout the definition of markers; *x* is the time series for one channel, 𝑘 is the time point, *N* is the number of time points in the segment, *C* is the number of recording channels, and *T* is the number of segments in the ictal period.

#### 2.3.1 Peak markers

**FIGURE 1:**
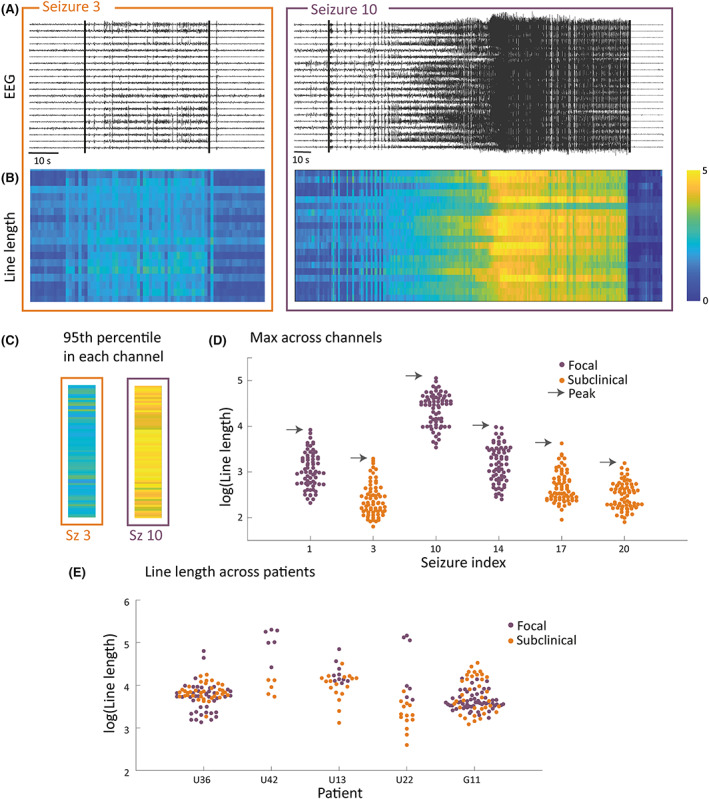
Visualizing the workflow for calculating peak markers for example patient U22. (A) Intracranial electroencephalographic (EEG) traces for a subclinical (orange) and focal (purple) seizure in an example patient, with a subsection of recording channels for visualization. (B) Heat maps of the line length marker in 1‐s epochs for seizures in A. (C) Ninety‐fifth percentile of line length measures for each channel across time. (D) Bee‐swarm representation of the same data as C, also for a few more example seizures in this patient. Gray arrows point to the maximum value across channels; this is the peak value for the seizure. (E) Log‐transformed peak line length values (maximum channel value across 95th percentiles), as indicated by gray arrows in D in five example patients; each data point represents a seizure. Sz, seizure.

The maximum level of activity in the ictal phase was estimated using peak markers of the iEEG features: line length,^23^, ^24^ energy,^25^ and band‐power^26^ (in *δ* [1–4 Hz], *θ* [4–8 Hz], *α* [8–13 Hz], *β* [13–30 Hz], low‐*γ* [30–60 Hz], and high‐*γ* [60–100 Hz] bands), each of which have previously been used within seizure detection algorithms.^21^, ^27^ Each seizure recording (Figure 1A) was separated into 1‐s epochs with no overlap from which each peak marker was calculated, resulting in eight *T* × *C* matrices (Figure 1B).

For each severity marker (i.e., each matrix) we first summarized markers across time; for each recording channel, the 95th percentile of each marker was calculated (Figure 1C). The maximum value across channels was then used as the estimated peak activity of the seizure (Figure 1D). As expected, markers differ across seizure types and patients (Figure 1E). Once summarized over time (by the 95th percentile of each channel across time) and across channels (maximum value), we log‐transformed the measures to normalize their distributions.

#### 2.3.2 Spatial markers

**FIGURE 2:**
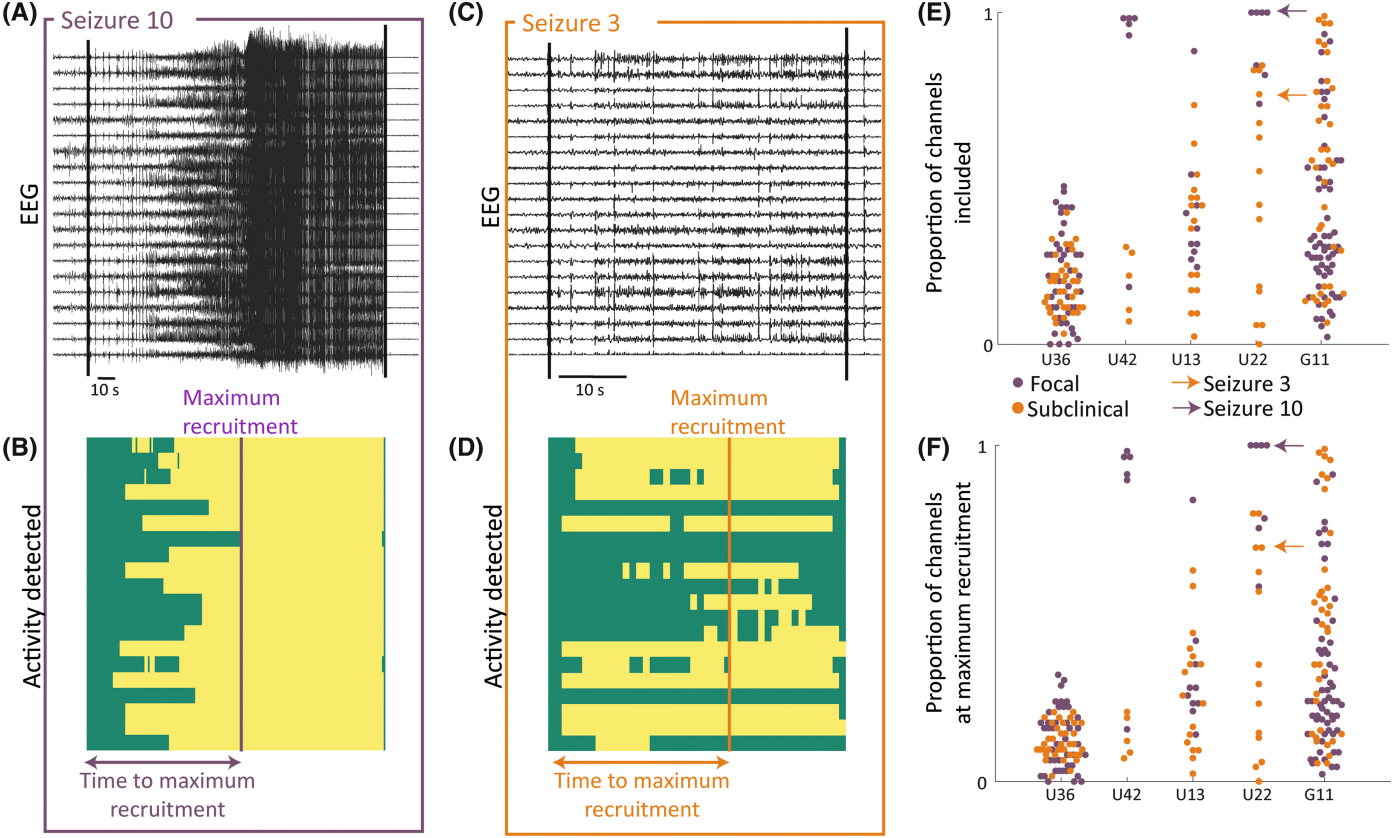
Visualizing spatial markers for example patient U22. (A, C) Intracranial electroencephalographic (EEG) traces of an example focal/subclinical seizure with a subset of recording channels. (B, D) Corresponding binary map of seizure imprint (yellow indicates seizure activity, green no seizure activity) across time in the same subset of channels as in A and C. (E) Swarm plot of the proportion of channels with seizure activity at any point in the seizure for all seizures in five example patients. (F) Swarm plot of the proportion of channels with seizure activity at the point of maximum recruitment for all seizures for five example patients.

The extent of the spread of ictal activity across recording channels was captured through spatial markers. For each channel, baseline (preictal) and ictal recordings were divided into 1‐s, nonoverlapping epochs, from which each of eight features (line length, energy, band‐powers in six frequency bands) were calculated. Seizure activity was algorithmically detected based on abnormality (median absolute deviation [MAD] scores) relative to the preictal period in each of the eight feature matrices. For each window per channel, an MAD score > 5 in any of the eight features suggested potential seizure activity. An additional step (see Appendix S3.4 for details) prevented spurious nonseizure activity from being detected (e.g., caused by noise or a short spike). This algorithm yielded a binary map identifying channels and time windows with seizure activity during the ictal period. We term this matrix the "imprint" of the seizure (see Figure 2A,C for EEGs and B,D for corresponding imprints).

Four markers were derived from the seizure imprint: the proportion of channels with seizure activity at any point in the ictal phase (Figure 2C, example patients), the proportion of channels with simultaneous seizure activity at the point of maximum recruitment (Figure 2D, example patients), the time taken from seizure onset to the time of maximum recruitment, and the proportion of the seizure duration taken to reach maximum recruitment.

#### 2.3.3 Suppression markers

**FIGURE 3:**
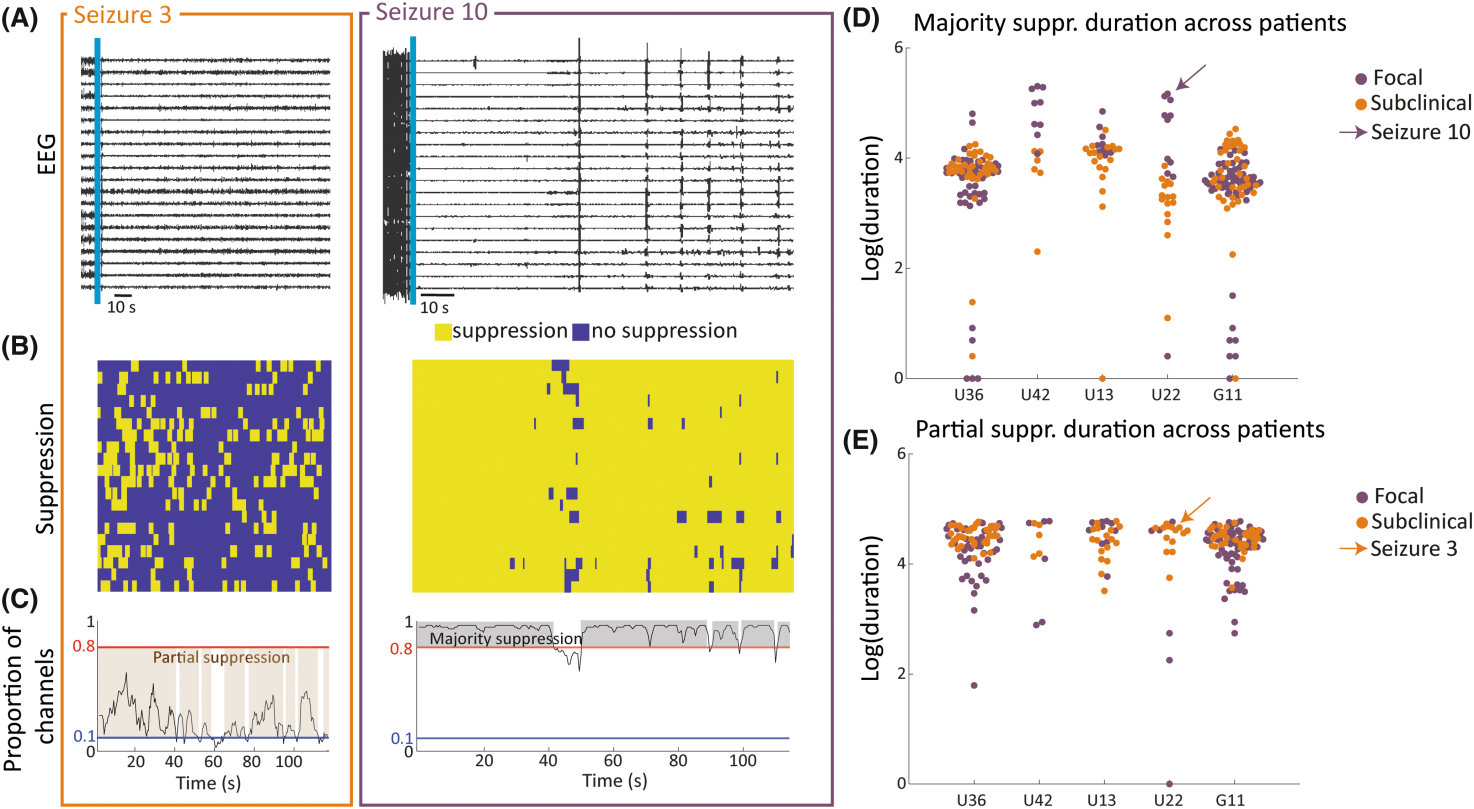
Visualizing suppression markers for example patient U22. (A) Intracranial electroencephalographic (EEG) traces of example subclinical (orange) and focal (purple) postictal segments in a subset of recording channels. (B) Corresponding binary maps of channels with suppression (<5% of preictal activity levels) in the same subset of recording channels. (C) Proportion of suppressed channels across 120 s of postictal activity. Segments of majority suppression and partial suppression are highlighted. (D) Swarm plot of (log‐transformed) majority suppression duration for all seizures for five example patients. (E) Swarm plot of (log‐transformed) partial suppression duration for all seizures for five example patients.

Duration and strength of postictal suppression was captured by our suppression markers. Signal range was computed in .5‐s nonoverlapping windows. For each channel, postictal ranges were compared against the distribution of preictal ranges. Ranges below the fifth percentile of the preictal range were labeled as suppressed (see Figure 3A for a postictal EEG and 3B for its corresponding suppression matrix). Periods of suppression were labeled as majority suppression or partial suppression based on the proportion of suppressed channels (Figure 3C). Durations of majority suppression and partial suppression (Figure 3D,E) were calculated using a 2.5‐s moving sum to account for short spikes of activity in suppressed segments. Further details are provided in Methods S3.5. The suppression duration was computed as the time following seizure offset with a 1‐s buffer. A third suppression marker, suppression strength, was defined as the median proportion of channels with suppression across the duration of the postictal recording. Although we analyzed 120 s of postictal activity, duration of suppression may have exceeded this 120 s.^9^ Therefore, suppression durations of 120 s in the following should be understood as "at least 120 s."

### 2.4 Statistical analysis

Statistical analyses were then performed in RStudio. Probability values were calculated for reference and visualization, not to stratify patients for further analyses.

#### 2.4.1 Validating markers against ILAE seizure classification

ILAE seizure classification^28^ was used as a validation for seizure severity. Our main analyses compared focal versus subclinical seizures and focal aware versus impaired awareness seizures; supplementary analyses are shown comparing focal versus FTBTC seizures. Performance of markers was assessed by how well they distinguish these seizure types. We applied two strategies for validation, across and within patients, to separately assess performance of markers in distinguishing clinically distinct seizure types.

##### Across patients

For each marker, three hierarchical logistic regression models were compared to assess marker and/or patient effects. Specifically, we created a model considering only random patient effects and two models considering both fixed marker effects and random patient effects (random intercept & random intercept and slope models). The fit of each model was assessed using Akaike information criterion, Bayesian information criterion, and deviance. Models with poor fit were deemed inadequate and removed. Assumptions of logistic regression models were checked for each model individually. The quality of each model as a classifier of seizure type was assessed through the area under the curve (AUC) for receiver operating characteristic curves with 100 decision thresholds. Performance was assessed based on AUC thresholds (AUC > .7 is acceptable, >.8 is excellent, and >.9 is outstanding).^29^ Supplementary analyses are shown for focal versus FTBTC seizures (Table S4.3) and focal versus subclinical seizures in TLE (Table S4.4) and eTLE (Table S4.5).

##### Within patients

Each marker's performance in distinguishing seizure types for each patient was assessed using two‐tailed Wilcoxon rank sum tests. Patients were included in within‐patient validation if they had a minimum of five seizures, with two or more seizures of each type. The distinction between markers of different seizure types was quantified using the effect size (*r*) calculated as:

$$r=\frac{Z}{\sqrt{N}}$$

where *Z* is the *Z*‐statistic and *N* is the total sample size. The *r* value was bounded between zero and one, with values closer to one indicating larger effects. It is common in the literature to consider .1 ≤ *r* < .3 as a small effect, .3 ≤ *r* < .5 as a moderate effect, and *r* ≥ .5 as a large effect.

#### 2.4.2 Circadian and longer term modulation of seizure severity

We additionally assessed circadian and longer term fluctuations in seizure severity. For individual patients, we assessed circadian fluctuations using rank circular–linear correlation^30^ using the *cylcop* R package.^31^ Probability values were calculated through a permutation test with 1000 permutations. Inclusion criteria were that patients must have 20 or more recorded seizures irrespective of the frequencies of each seizure type. This threshold was chosen based on performance of circular–linear correlation on simulated data with varied sample sizes and noise. Long‐term fluctuations in severity were assessed using Spearman rank correlation between markers and the time since first recorded seizure.

### 2.5 Code and data availability

The analysis code and data are available on Zenodo.org (DOI: 10.5281/zenodo.7575874). The expandable library of severity markers is already available on GitHub (https://github.com/cnnp‐lab/seizure_severity_library), and we invite contributions from the community.

## 3 RESULTS

We computed each of the 16 proposed seizure severity EEG markers for all 1009 recorded seizures. We first validated each marker by assessing performance in distinguishing different ILAE classification both across all patient seizures and within each patient. However, we envisage additional uses of this library and, as an example, demonstrate its potential ability to detect fluctuations in seizure severity over time.

### 3.1 Severity markers distinguish between ILAE clinical seizure types across patients and seizures

**FIGURE 4:**
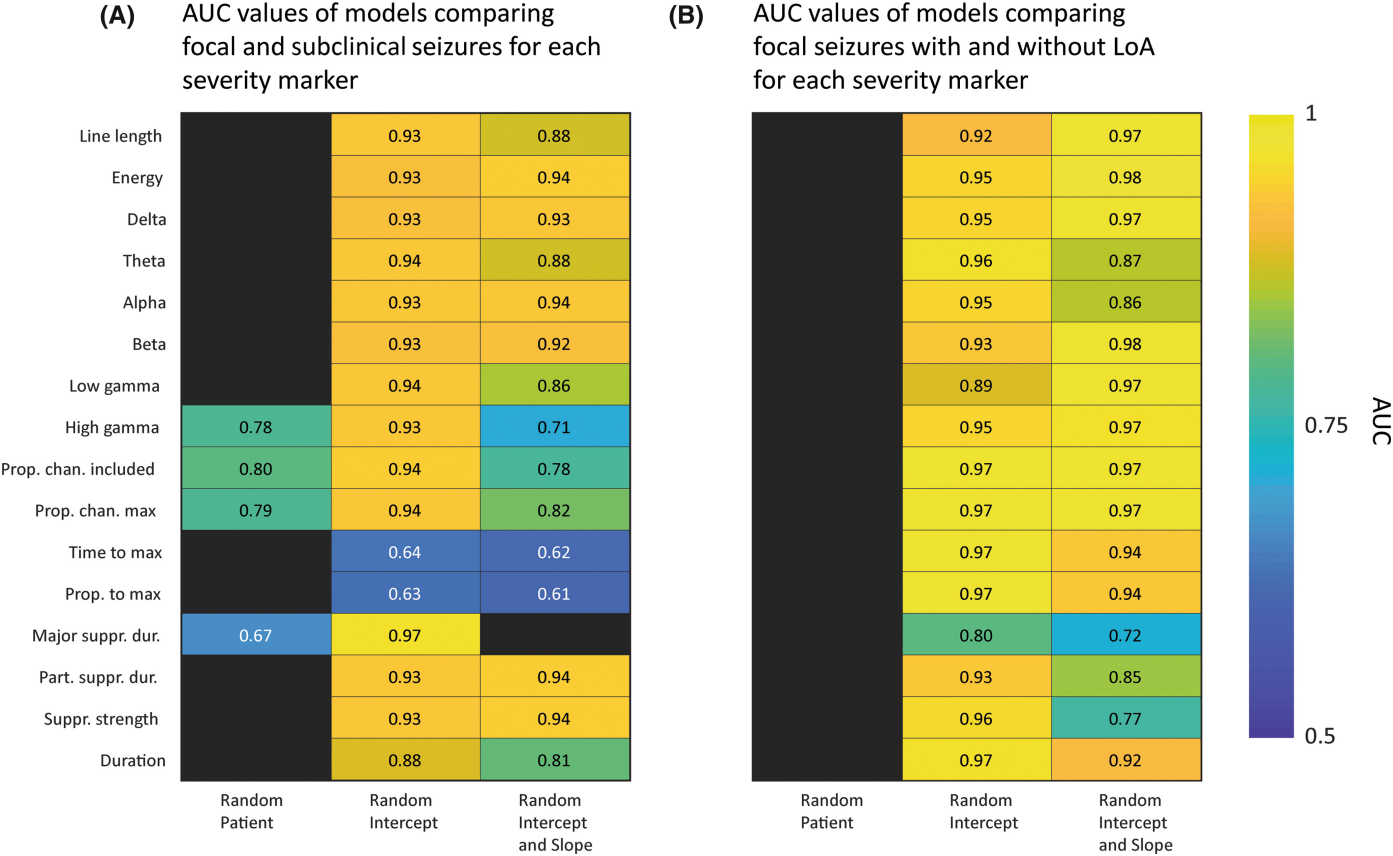
Validating markers against International League against Epilepsy classification across patients. (A) Heat map of area under the curve (AUC) values for hierarchical logistic regression models comparing focal and subclinical seizures. (B) Heat map of AUC values for hierarchical logistic regression models comparing focal seizures with and without loss of awareness (LoA).

To validate our markers, we assessed their ability to distinguish focal versus subclinical seizures and focal seizures with and without impaired awareness across patients. Specifically, for each of the 16 markers, we compared seizure types across all patients using hierarchical mixed effects logistic regression models. Figure 4 displays the AUC values obtained for each model in all markers when comparing focal versus subclinical (A) and focal seizures with and without impaired awareness (B). There were clear patient differences in the marker values; however, the majority of models created with only patient effects were unacceptable classifiers (AUC < .7 or model assumptions not met), suggesting that between‐patient differences alone did not account for differences between focal and subclinical seizures. In contrast, 14 severity markers yielded excellent classifier performance with random intercept models or random intercept and slope models. As seizure duration is often used to assess seizure severity,^22^ we compared the performance of each marker against the performance of duration in distinguishing seizure types (see Figure S4.1C,D) using a bootstrapping procedure (see Methods S3.6). When comparing focal versus subclinical seizures, observed AUC values for all markers (except time and proportion of seizure to maximum recruitment) were larger than most of the distribution of AUC values for seizure duration. When comparing focal seizures with and without impaired awareness, all peak markers except theta and alpha band‐powers, and all spatial markers outperformed seizure duration.

Appendix S4.1 shows additional results comparing focal versus FTBTC seizures and comparing focal versus subclinical seizures in TLE and eTLE. When comparing focal versus FTBTC seizures, all markers created excellent or outstanding classifiers through random intercept models. We further subdivided patients into those with TLE and those with eTLE, from which we repeated across‐patient analyses for focal versus subclinical seizures. When separating patients into TLE and eTLE only, sample sizes were 360 and 595 seizures, respectively. For TLE patients, five markers showed excellent or outstanding performance in random intercept models (Table S4.4). For eTLE patients, all markers had excellent or outstanding performance in random intercept and/or random intercept and slope models (Table S4.5).

### 3.2 Severity markers distinguish between ILAE clinical seizure types within patients

We next validated our markers by quantifying distinctions between ILAE seizure types within individual patients. We analyzed effect sizes between seizure types using Wilcoxon rank sum test r‐values. Using our inclusion criteria, we could compare focal and subclinical seizures in 15 patients. Patients included in this analysis did not differ in demographics (sex, age, disease duration, and epilepsy diagnosis) relative to the entire cohort. Majority suppression duration could not be validated, as many patients did not have sufficient seizures with periods of majority suppression.

**FIGURE 5:**
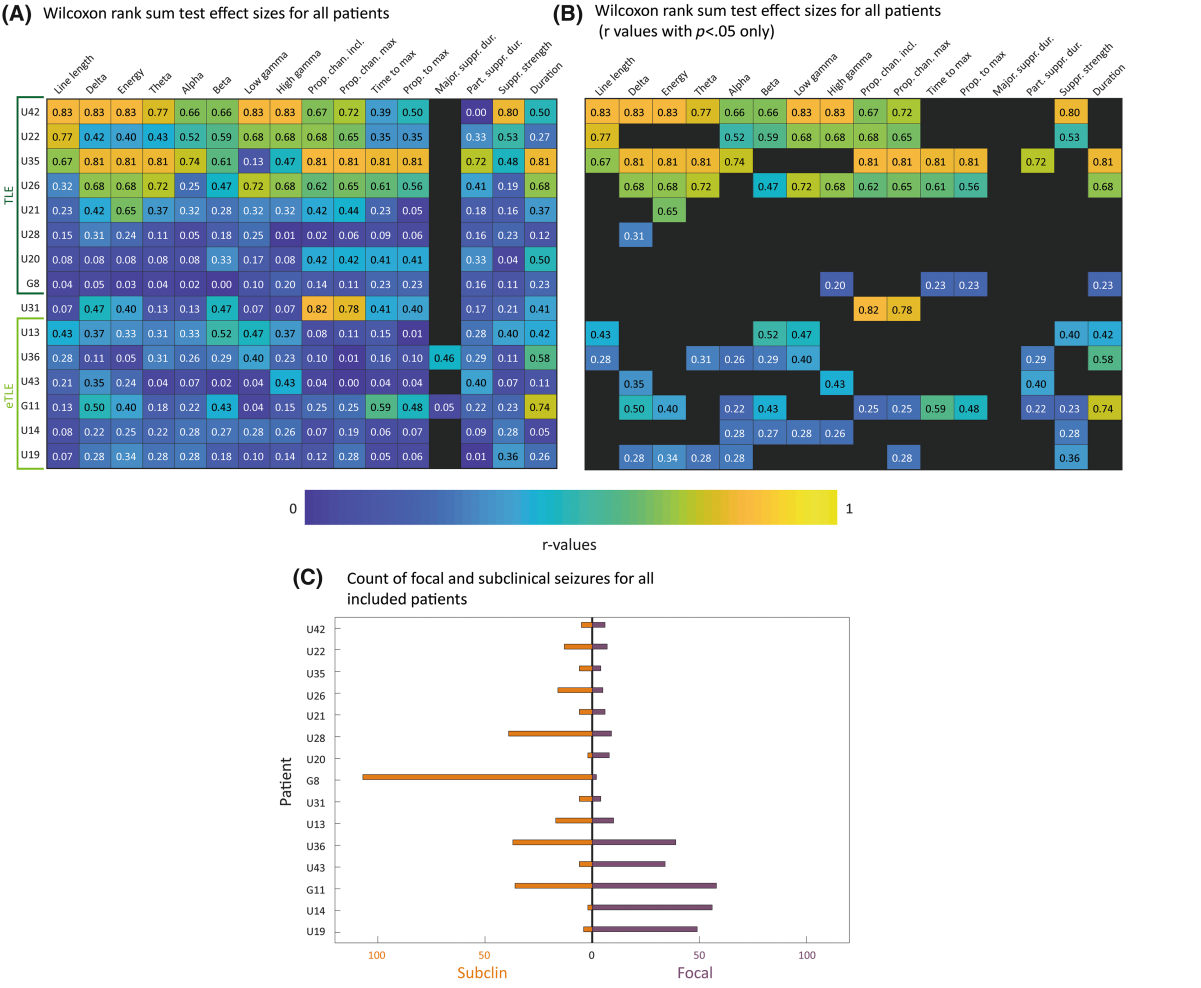
Validating markers against International League against Epilepsy (ILAE) classification on a within‐patient basis. (A) Wilcoxon rank sum test *r*‐values obtained through comparing focal and subclinical seizures. Each row is a patient, and each column is a marker. Patients were sorted by descending *r*‐values within the temporal lobe epilepsy (TLE) and extra‐temporal lobe epilepsy (eTLE) groups. (B) Same as in panel A, filtered by *p* < .05. (C) Paired bar chart displaying counts of focal and subclinical seizures for each patient included in within‐patient validation.

Moderate to large effects (*r* > .3, *p* < .05) in three or more markers were found for eight of the 15 included patients (53.3%). The heat maps of *r*‐values are shown in Figure 5A,B. Figure 5B shows a heat map of r‐values only where *p* < .05. The number of focal and subclinical seizures recorded per patient varied (see Figure 5C). Effects were notably higher in four patients, all of whom were TLE patients, supporting that performance of markers is likely patient‐specific. We investigated the effect of various other patient metadata (sex, TLE/eTLE, surgical outcome, disease duration, age, number of recording channels, and number of recorded seizures) on marker performance (see Table S4.6). Most notably, there was a large effect between spatial markers for patients with TLE compared to eTLE, but none of the other patient features showed consistent or noteworthy effects. Comparing performance of our markers against seizure duration, in five patients (33%), duration alone was not a useful marker of seizure severity (*r* < .3, *p* > .05). However, in each of these patients, at least three other markers were useful (*r* > .3, *p* < .05) in distinguishing focal and subclinical seizures.

### 3.3 Seizure severity changes across different timescales

**FIGURE 6:**
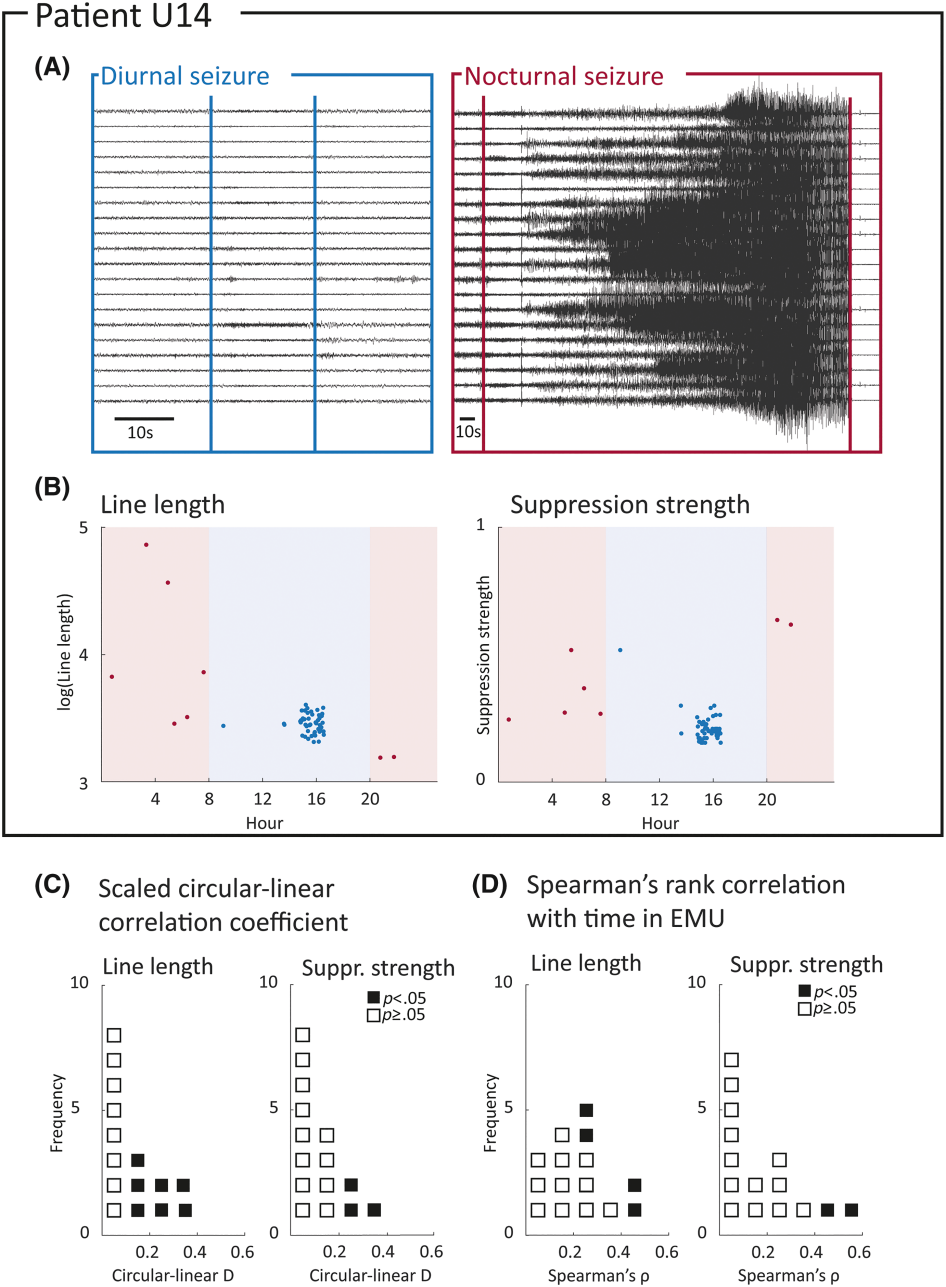
Detecting circadian and longer term modulation of seizure severity. (A) Intracranial electroencephalographic recordings for a daytime (blue) and night‐time (pink) seizure from example patient U14. (B) Plot of marker against time of day for line length and postictal suppression strength. Pink background indicates evening/night, whereas blue background indicates daytime. (C) Dot plot of scaled circular–linear correlation coefficients between markers and time of day across included patients. Probability values < .05 obtained through permutation tests are highlighted in black. (D) Dot plot of absolute Spearman rank correlation coefficient between markers and time in epilepsy monitoring unit (EMU) across included patients. Correlations with *p*‐values < .05 are highlighted in black.

Finally, we used our markers to capture fluctuations in seizure severity on circadian and longer timescales in 15 patients. Figure 6A shows example daytime and nighttime seizure iEEG traces from the same patient, U14. In U14, seizures occurring at different times of day appeared to have different characteristics; for example, line length and suppression strength differences are higher in nocturnal seizures (Figure 6B,C). The association between these markers and seizure times was measured using circular–linear correlation.^30^ Eight patients (66.7%) had correlations with *ρ* > .2 and *p* < .05 for at least three markers.

We additionally asked whether our severity markers also changed over the span of each patient's recording. Figure 6D shows the absolute Spearman rank correlation between two example markers and the time of each seizure relative to the start of the recording. This measure captures the strength, but not the direction, between marker values and the time of seizure occurrence. In eight of 15 patients (53.3%), at least three markers had correlations with *ρ* > .3 and *p* < .05 with the amount of time elapsed since the start of the recording. Correlation coefficients for all markers are shown in Tables S4.7–S4.10. Moderate to strong correlations can be seen in a wide range of markers and patients; thus, we conclude that circadian and longer term changes in EEG severity can be detected in the majority of patients.

We were limited by the time spent in the EMU; therefore, our findings on modulation are proof‐of‐concept. These results should be interpreted as evidence that our markers could be used to capture fluctuations in severity.

## 4 DISCUSSION

We evaluated 16 objective quantitative markers of seizure severity derived from iEEG recordings of patients with refractory focal epilepsy. Our goal was to offer a collection of markers that can be used as output measures for clinical trials, tracking fluctuations in seizure severity, or other applications. Our results demonstrated that almost all severity markers could distinguish focal versus subclinical seizures across our cohort of 63 patients. Importantly, marker performance was patient‐specific, indicating that different groups of patients are best evaluated with a subset of our proposed markers; thus, our approach of providing a severity library for future work to draw from is an important contribution. We also found that severity fluctuated on circadian and longer term timescales in a patient‐specific manner, supporting the use of EEG‐based severity markers to investigate temporal modulation of seizure severity. Our work may therefore also facilitate personalized, time‐adaptive treatments or enhance our understanding of the chronobiology of seizures.

Existing scales of seizure severity have been used as outcome measures in clinical trials.^32^, ^33^, ^34^, ^35^ However, scales depend on patients' ability to recall seizures over weeks,^4^, ^6^ leading to concern over their reliability. Many scales also focus on patient risk rather than objective severity. For example, the NHS3 stipulates that seizures occurring in bed are automatically scored zero for falls, potentially underestimating their electrographic and neurobiological severity. No existing scales assess individual seizure severity in an objective quantitative manner, making small changes in severity difficult to capture. Our library of quantitative EEG markers addresses these limitations, providing a complementary approach for measuring and understanding seizure severity.

Our approach to validating our markers was to compare two seizure types that have obvious distinctions in terms of their neurobiological and symptomatic severity: namely, subclinical versus focal seizures. The proportion of subclinical versus focal seizures within these data (323 vs. 656) agrees with previous literature,^36^ suggesting that our seizure type labels are not biased. Previous literature suggests that subclinical and focal seizures have different EEG features,^37^ even within the same patient,^36^ thus making it a good standard to compare to. However, our proof‐of‐principle validation against seizure type is only one of many possible standards; future work could test other standards that are tailored to the research question.

One main finding of this work was that the performance of seizure severity markers derived from iEEG recordings is highly patient‐specific. Peak markers tended to perform well, as did some spatial markers (proportions of channels measures). The remaining markers varied in their performance, even among patients with better distinctions based on other markers. Results suggest that spatial markers have the highest performance in distinguishing focal seizures with and without impaired awareness. We suggest testing the entire library of markers for each new patient to determine which, if any, are the most appropriate for the desired application.

Different aspects of seizure severity have been repeatedly reported to follow circadian, sleep/wake, and longer timescale modulations. For example, secondary generalization and postictal suppression occur more often in seizures arising from sleep.^11^, ^13^, ^14^ Subclinical seizures are also reported to follow a circadian pattern.^15^ Recent studies also reported modulations at circadian and longer timescales within many patients in terms of seizure electrographic evolutions^38^, ^39^ and other seizure properties.^40^ In agreement with previous literature, we found evidence that EEG‐based seizure severity markers are modulated on circadian and longer timescales, although the effect size of the modulation is patient‐specific and weak in some patients. We suggest that, similar to previous work,^39^ capturing data of the potential modulations and directly relating those to the severity markers in a multivariate model may be insightful.

### 4.1 Limitations and future work

The patients included in this study are presurgical candidates with refractory focal epilepsy; therefore, our library needs to be expanded and tested in other epilepsy syndromes. The use of iEEG allows for good signal quality but does not capture activity beyond a small part of the brain. Electrode placement was determined by clinical need, and therefore the location of electrodes varied across patients. This variability means that spatial markers do not capture the same information in different patients, and thus hierarchical statistical approaches are needed to compare markers across patients. Future work could use simultaneous scalp EEG and iEEG to validate markers of spread based on iEEG in different anatomical regions. Within this work, spatial markers based on activity in regions of interest (ROIs) rather than individual channels was considered; unfortunately, electrode location was not available for all patients. We opted to maintain our channel‐based spatial and suppression markers to maintain our sample size. Further research including a larger cohort with available electrode location information is required to confirm that spatial markers derived from ROIs could be used to capture seizure severity. Our methods could be extended to subscalp EEG with some alterations to account for lower spatial coverage. Although the lower coverage presents a challenge, previous studies suggest encouraging findings. For example, Parvez and Paul^41^ predicted seizure occurrence using only six recording channels per patient. Furthermore, recordings from only 16 locations on the surface of the brain captured critical slowing,^42^ giving evidence that alterations in EEG around seizures can be captured with few electrodes. Extension of our library to scalp EEG and other modalities is planned, and with our open code base on GitHub, we welcome contributions from the community.

As recordings took place in EMUs, patients were also under nonnormal conditions during recordings; antiseizure medications are often tapered, and patients are potentially under an increased amount of stress. Future work might use continuous recordings to capture the full range of interictal brain dynamics to better estimate spatial and suppression properties of seizures. Future work should also investigate the three‐way relationship between severity markers, seizure type, and circadian influences. Furthermore, electrographic activity can fluctuate for weeks following electrode implantation^43^; however, the preictal baseline that we applied for spatial and suppression markers may render those markers less sensitive to such fluctuations. Future work needs to disentangle the biological, technological, and pathological influences on EEG biomarkers; this remains an open challenge for various applications. Such fluctuations may have influenced the results of this work, especially in modulation analyses. Regardless, our results remain meaningful as a proof‐of‐concept that our markers can be used to detect fluctuations in ictal electrographic activity and, by extension, seizure severity.

## 5 CONCLUSIONS

In conclusion, we propose 16 EEG markers of seizure severity that can be used to complement existing measures. Most markers were validated against ILAE classification on an across‐patient basis. Marker performance, as measured by their ability to distinguish seizure types and capture fluctuations in seizure severity, is strongly patient‐specific. We also detected circadian and longer timescale fluctuations in seizure severity, which may be relevant for a range of applications including capturing treatment response and seizure forecasting.^44^, ^45^, ^46^ Our library therefore contributes to ongoing efforts in characterizing seizures over time, seizure prediction, and generally designing novel, personalized treatment plans that manage and mitigate severe seizures.

## AUTHOR CONTRIBUTIONS

Conceptualization: Sarah J. Gascoigne, Leonard Waldmann, Gabrielle M. Schroeder, Mariella Panagiotopoulou, Peter N. Taylor, and Yujiang Wang. Methodology: Sarah J. Gascoigne, Leonard Waldmann, Gabrielle M. Schroeder, Mariella Panagiotopoulou, Christoforos Papasavvas, and Yujiang Wang. Software/validation: Leonard Waldmann and Yujiang Wang. Formal analysis: Sarah J. Gascoigne, Mariella Panagiotopoulou, and Yujiang Wang. Resources: Fahmida Chowdhury, Alison Cronie, Beate Diehl, John S. Duncan, Jennifer Falconer, Veronica Leach, and Shona Livingstone. Data curation: Jess Blickwedel, Ryan Faulder, Gabrielle M. Schroeder, and Yujiang Wang. Writing: Sarah J. Gascoigne, Leonard Waldmann, Gabrielle M. Schroeder, Mariella Panagiotopoulou, John S. Duncan, Christoforos Papasavvas, Kevin Wilson, Peter N. Taylor, and Yujiang Wang. Supervision: Yu Guan, Rhys H. Thomas, Kevin Wilson, Peter N. Taylor, and Yujiang Wang.

## CONFLICT OF INTEREST

None of the authors has any conflict of interest to disclose.

## Supporting information


Appendix S1
