# Supplementary material for: A library of quantitative markers of seizure severity
Source: Epilepsia. 2023 Feb 17;64(4):1074–86. doi: 10.1111/epi.17525 (PMC10952709; doi:10.1111/epi.17525)
Supplement: Supplementary file 1 — Appendix S1 [file EPI-64-1074-s001.pdf]

Supplementary materials: A library of quantitative markers of seizure severity

S1 Glossary of acronyms

Clinical terms

ASMs: Anti-seizure medications
EEG: Electroencephalography
EMU: Epilepsy Monitoring Unit
eTLE: Extratemporal lobe epilepsy
FTBTC: Focal to bilateral tonic clonic (seizure)
iEEG: Intracranial EEG
ILAE: International League Against Epilepsy
LSSS: Liverpool seizure severity scale (Baker et al., 1991)
NHS3: National Hospital Seizure Severity Scale (O'Donoghue et al., 1996)
SSQ: Seizure Severity Questionnaire (Cramer et al., 2002)
TLE: Temporal lobe epilepsy

Statistical terms

AUC: Area under the curve
CAR: Common average reference
MAD: Median absolute deviation
ROC: Receiver operating characteristic

S2 Patient Metadata

We retrospectively analysed iEEG recordings from a cohort of 63 patients undergoing presurgical evaluation for refractory focal epilepsy. All patients had electrodes surgically implanted as grids and/or strips.

- **Age (yrs):** patient age in years (Median = 29, SD = 7.072).
- **Sex:** patient sex (30 male, 32 female, 1 unknown)
- **Disease duration (yrs):** Time between epilepsy diagnosis and recording in years (Median = 22, SD = 8.581).
- **Diagnosis:** Purported lobe of onset of the patient's seizures, based on clinical findings (32 TLE, 25 eTLE, 6 unknown). For this analysis, diagnoses were categorised as TLE or eTLE.

The number of subclinical, focal and FTBTC seizures is listed in Supplementary Table S2.1; the seizure types experienced by each individual are listed in Supplementary Table S2.2.

Seizure type	Number of patients (n=1009)
Focal impaired awareness	176
Focal aware	232
Focal (awareness unknown)	248
Subclinical	323
FTBTC	6
Unknown	24

Table S2.1: Table of counts of seizure types in dataset.

Seizure type	Number of patients ($n = 63$)
Focal only	29
Subclinical only	2
FTBTC only	2
Focal + Subclinical	23
Focal + FTBTC	2
Focal + Subclinical + FTBTC	2
Unknown	3

Table S2.2: Table of distribution of seizure types for individual patients.

S3 Supplementary Methods

S3.1 Noise Detection

Prior to computation of markers and subsequent analysis, each iEEG recording was assessed for noise. Muscle movements and eye blinks were not concerning here as iEEG electrodes are placed directly onto or into the brain and thus are not susceptible to such sources of noise. However, this data was screened for noise from other potential sources. Line noise was removed using a notch filter at 50Hz and 100Hz (with 2Hz windows).

The preictal segment was used to compute a baseline of electrographic activity, which was used in detection of seizure activity and postictal suppression. For more reliable estimates, noise was algorithmically detected as follows:

1. Raw iEEG time series MAD scored based on variance and min-max range for each channel independently
2. MAD>16 labelled as 'outlier' - channel is noisy
3. 'Noisy' channels removed
4. iEEG time series common average referenced (CAR)
5. MAD>16 labelled as 'outlier' - channel is noisy
6. 'Noisy' channels removed
7. 1Hz high-pass Butterworth 4th order filter used to remove any slow trends
8. Repeat the process with a less lenient threshold of MAD>12.
9. Visual check

Visual checks were performed to ensure that noise detected was, indeed, noise and to identify potential noise that was not detected. Following this, markers of seizure severity were computed. Noise in the ictal segment was visually assessed using iEEG traces and power spectral density plots - noisy channels were removed from all recordings. We did not seek or remove noise impacting only the postictal segment.

S3.2 Seizure Severity Markers

We calculated 16 markers of seizure severity based on iEEG recordings. Each marker captures a different aspect of seizure severity; descriptions of the markers and relevant equations are listed in Table S3.1. Our library of objective seizure severity markers has three main branches: peak, spatial, and suppression markers.

Branch	Marker	Description	Equation
Peak	Line length	A measure of the **complexity** of iEEG signals	$\frac{1}{N}\sum_{k=1}^{N-1}\lvert x_{k-1}-x_k\rvert$
	Energy	**Relative energy** across all frequency bands	$\sum_{k=1}^{N}(x_k-\bar{x})^2$
	δ band-power	Power in **1-4Hz** frequency band	
	θ band-power	Power in **4-8Hz** frequency band	
	α band-power	Power in **8-13Hz** frequency band	
	β band-power	Power in **13-30Hz** frequency band	
	Low γ band-power	Power in **30-60Hz** frequency band	
	High γ band-power	Power in **60-100Hz** frequency band	
Spatial	Proportion of channels included	Proportion of channels with seizure activity at **any point** in the ictal period	
	Proportion of channels at the point of maximum concurrent activity	Proportion of channels with seizure activity at the point of **maximum concurrent** activity	
	Time to max	Time to **maximum concurrent activity** (in seconds)	
	Prop to max	The **proportion of seizure duration** to point of maximum concurrent activity	
Suppression	Majority suppression duration	Time (in seconds) post-ictally with suppression detected in $\geq$ **80%** of recording channels	
	Partial suppression duration	Time (in seconds) post-ictally with suppression detected in $\geq$**10%** and <**80%** of recording channels	
	Suppression strength	Median proportion of channels with suppression across the duration of the post-ictal recording	
N/A	Duration	**Duration** of the ictal period in seconds (based on visual inspection of iEEG)	

Table S3.1: Table of 16 iEEG-based seizure severity markers proposed within this paper

S3.3 Peak markers

Signal complexity was captured using line length(Olsen et al., 1994), calculated as:

$$\frac{1}{N}\sum_{k=1}^{N}|x_{k-1}-x_k| \text{ (Esteller et al., 2004)}$$

The strength of the EEG signal was captured by calculating the signal's energy:

$$\sum_{k=1}^{N}(x_k-\bar{x})^2 \text{ (Hamad et al., 2016)}$$

where $\bar{x}$ is the mean of the time series.

For each severity marker, we first summarised markers across time; for each recording channel, the 95th percentile of each marker was calculated. We selected the 95th percentile rather than the maximum value to reduce the risk of capturing outlier values which may not have been representative of true seizure activity. The maximum value from this array was then used as the estimated peak activity of the seizure. Each of the peak markers was log-transformed to normalise their distributions. As expected, markers differed across seizure types and patients.

S3.4 Spatial markers

Spatial markers were designed to capture the extent of spread of ictal activity across recording channels. Seizure activity was detected using the eight features (line length, energy, band-power in six frequency bands) discussed above. Ictal changes in these features were compared to pre-ictal EEG. For each channel, baseline (pre-ictal) and ictal recordings were split into 1 second, non-overlapping windows. Each of the eight features were calculated for all windows. These computations yielded a baseline distribution of values for each feature and channel. We then scored ictal feature values relative to the baseline distribution to derive if and when a channel was invaded by seizure activity.

In detail, the pre-ictal baseline distribution was obtained for each feature and each channel following an automated rejection of pre-ictal spikes or artefacts. We achieved this by removing outliers (in any feature) from the distribution with median absolute deviance (MAD) greater than five. To score each ictal window to the baseline, we used the MAD score, which scores a given observation in terms of the median absolute deviation from the median. We chose MAD scores over z-scoring as this method is more robust to outliers.

Finally, to derive if any given window in a channel displays seizure activity, we obtained the maximum MAD score across all eight features, effectively measuring if the EEG activity deviated from baseline in any feature. Any window with a maximum MAD score greater than five was deemed as potentially displaying seizure activity. This step yields a binary matrix (of size number of channels by number of time windows) indicating potential seizure activity. To avoid detection of spurious non-seizure activity (e.g. caused by a brief noise or spike), we further validated the binary matrix with a sliding window approach. A symmetric moving sum of length $2\times\tau+1$ was

applied to the binary matrix. If the sum within each sliding window exceeded τ, this window was labelled as having seizure activity. In other words, a channel and time window is deemed to contain seizure activity only if in its temporal vicinity (τ) more than half of windows also showed potential seizure activity. We calculated τ as 10% of the seizure duration (d). Durations varied from five to 600 seconds in our data; therefore, we bound τ between two and five seconds to prevent extreme window lengths:

$$\tau = \begin{cases} 2 & \text{if } d \times 0.1 < 2 \\ d \times 0.1 & \text{if } 2 \leq d \times 0.1 \leq 5 \\ 5, & \text{if } d \times 0.1 > 5 \end{cases}$$

In this work, we combined eight markers to capture the spread of seizure activity. For each recording channel, the scale of abnormality compared to the preictal baseline was calculated in each marker. This list of markers is non-exhaustive, it is possible to increase the number of biomarkers included in this algorithm. Future work could expand our list of features (e.g., HFO activity), and we welcome contributions to the library by the community.

S3.5 Suppression markers

Duration and strength of post-ictal suppression was captured by our suppression markers. Signal range was computed as $x_{max} - x_{min}$ in 0.5-second non-overlapping windows. Periods of suppression (calculated in 0.5 second windows) were labelled as majority or partial suppression based on the proportion of suppressed channels: majority suppression was defined as suppression present in over 80% of recording channels, while partial suppression was defined as suppression between 10% and 80% of the channels. Duration of majority and partial suppression were calculated using a 2.5-second moving sum to account for short spikes of activity in suppressed segments. I.e. if a short spike of activity lasted for less than 2.5 seconds, those time points would still be labelled as suppressed. The duration was computed as the time following seizure offset with a one-second buffer.

The proportion of seizures with majority suppression differ across seizure types, as reported in Table S3.2. As expected, the proportion of seizures with majority suppression increases with the increasing severity of seizure types. Most seizures were found to have post-ictal partial suppression, the only seizures without such suppression had majority suppression for the entire postictal period. The partial suppression marker is likely to suggest suppression in seizures as the threshold for suppression is 5% of the preictal mean; therefore, with multiple channels and 120-time epochs per channel many instances of suppression will be highlighted by chance. We invite future work to adjust our threshold of 5%, for example a threshold of 1% of the baseline will encounter fewer false positives.

Seizure type	Subclinical	Focal	FTBTC
Majority suppression	31	172	6
No majority suppression	292	487	0
Proportion of seizures with majority suppression	9.6%	26.1%	100%

Table S3.2: Table of counts of seizures with and without majority suppression by seizure type.

S3.6 Comparison of marker performance against seizure duration

As seizure duration is often used to capture severity of seizure severity (Beniczky et al., 2020), we performed a bootstrapping procedure to compare the across-patient performance of all other markers to seizure duration. We computed a distribution of AUC values based on models created using seizure duration as a marker of seizure severity as follows:

1. Create a re-sampled data set by drawing with replacement. For each seizure type in each patient, we re-sampled observations, thereby maintaining the total sample size as well as the number of seizures of each type for each patient.

2. Created random intercept and random intercept and slope models based on seizure duration, and calculated AUC values for these models.

3. Steps 1 and 2 were repeated until 1000 non-Nan AUC values for each model type were computed. The two resultant distributions represented the performance of seizure duration in across-patient analyses.

4. Calculate the proportion of the duration distribution below the observed AUC for each marker.

Each AUC value for the remaining markers were compared the distribution of duration AUC values, the calculated proportion values was used to approximate the scale of improvement beyond the performance of seizure duration. Values were bounded between 0 and 1, with values close to 0 suggesting that duration was the superior marker and values close to 1 suggesting that the alternative marker was superior to seizure duration. The smaller sample size when comparing focal seizures with and without impaired awareness should be noted here, results should be interpreted with caution.

S4 Assessing Marker Performance

There is currently no gold standard for assessing the severity of epileptic seizures, therefore we validated each of our markers by assessing their performance in classifying seizure types with known differences in severity. Markers were calculated for subclinical (least severe), focal, and FTBTC (most severe) seizures. Markers were validated across all patients and on an individual patient basis.

S4.1 Across patients

For each model, four hierarchical logistic regression models were created to validate markers. Four models were created for each marker:

- **Random patient (RP) effects**: Only patient effects are included in the model. This model was used to determine if the distinction between seizure types is driven by patient differences.
- **Fixed marker and random patient effects (random intercept) (RI)**: Here, both fixed marker effects and random patient effects (in the form of random intercepts). This model captures the performance of markers, whilst considering the difference in marker values across patients.
- **Fixed marker and random patient effects (random intercept and slope) (RIS)**: Here, both fixed marker effects and random patient effects (in the form of random intercepts and slopes). This model captures the performance of markers, whilst considering the difference in marker values and changes in marker values between seizure types across patients.

The performance of each marker was assessed using the area under the curve (AUC) receiver operator curve (ROC). An AUC value of 0.7 or greater was considered acceptable, above 0.8 was considered excellent and above 0.9 was outstanding (Mandrekar, 2010). Supplementary Table S4.1 displays the AUC values for each model type in each marker comparing subclinical *vs.* focal seizures. Models with poor fit to the data, as shown by large deviance values, were removed from analysis (AUC is shown here as NaN).

When comparing focal aware and impaired awareness seizures, there were clear patient differences in the marker values; however, the majority of models created with only patient effects were unacceptable classifiers (AUC < 0.7) or poor fit to the data, suggesting that between-patient differences alone did not account for differences between focal aware and impaired awareness seizures. In contrast, 14 severity markers yielded excellent classifier performance with random intercept models or random intercept and slope models. Supplementary Table S4.2 lists AUC values. In validating markers against focal and FTBTC seizure classifications, all markers created excellent or outstanding classifiers using random intercept models. Supplementary Table S4.3 lists AUC values. However, the sample size of FTBTC seizures was very small (n=6), therefore the results of this analysis are indicative of good performance but further testing on a larger data set is required.

We further divided patients into patients with TLE and eTLE to determine if the performance of markers (focal *vs.* subclinical) was impacted by the lobe in which seizures began. Tables S4.4 and S4.5 display AUC values for hierarchical logistic regression models for TLE and eTLE patients, respectively.

Comparing AUC values from all patients and TLE and eTLE patients separately, these results

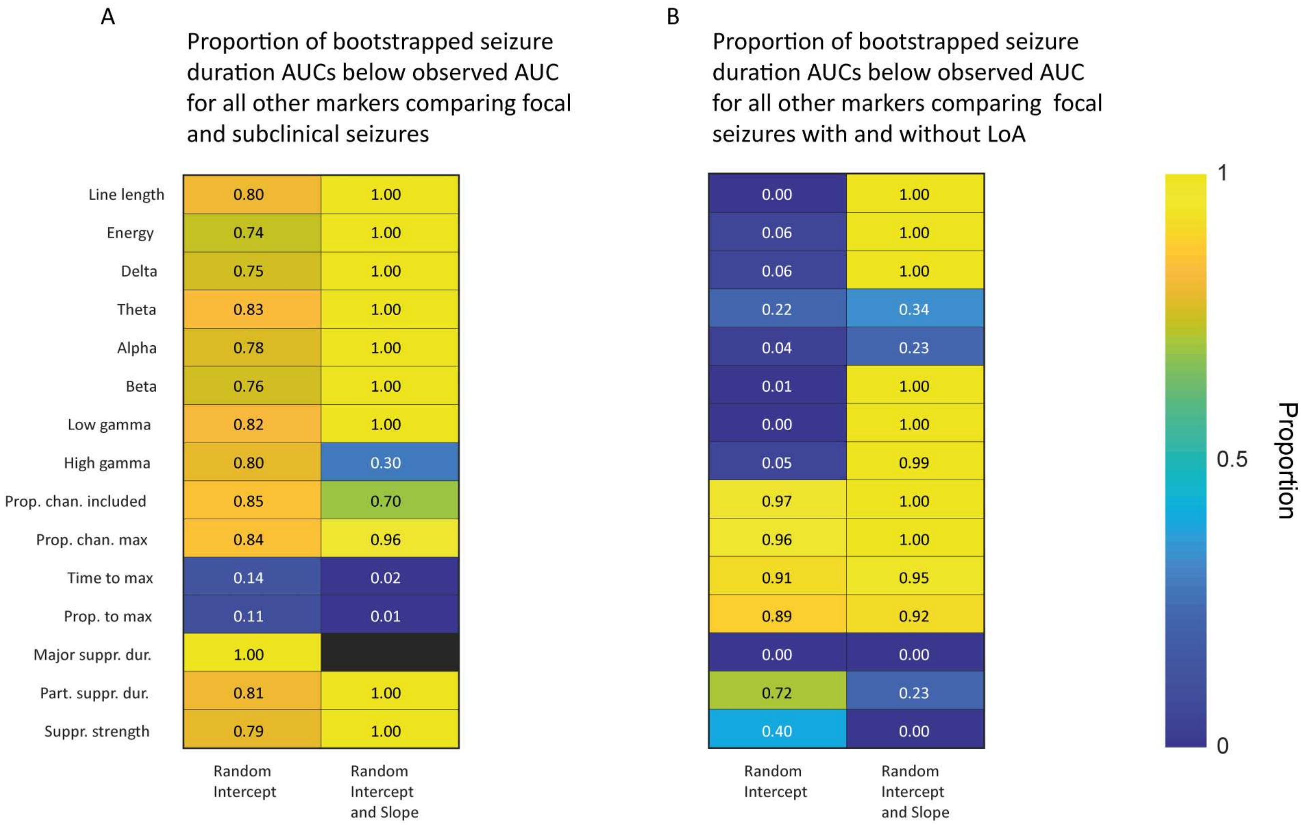

Figure S4.1: Validating markers against ILAE classification across patients.
A) Heat-map representing the proportion of the bootstrapped duration distribution below the observed AUC for all other markers for random intercept and random intercept and slope models comparing focal *vs.* subclinical seizures. B) Heat-map representing the proportion of the bootstrapped duration distribution below the observed AUC for all other markers for random intercept and random intercept and slope models comparing focal seizures with and without impaired awareness.

**Marker**	RP	RI	RIS
Line length	NaN	**0.934**	0.881
Energy	NaN	**0.926**	**0.938**
δ band-power	NaN	**0.928**	**0.934**
θ band-power	NaN	**0.937**	0.876
α band-power	NaN	**0.930**	**0.936**
β band-power	NaN	**0.929**	**0.923**
Low-γ band-power	NaN	**0.935**	0.859
High-γ band-power	0.785	**0.932**	0.706
Prop. chan. included	0.800	**0.938**	0.777
Prop. chan. at MR	0.788	**0.937**	0.823
Time to MR	NaN	0.635	0.620
Prop. of seizure to MR	NaN	0.629	0.612
Major. suppr. duration	0.668	**0.968**	NaN
Part. suppr. duration	NaN	**0.935**	**0.940**
Suppr. strength	NaN	**0.931**	**0.940**
Duration	NaN	0.884	0.812

Table S4.1: Table of area under the curve (AUC) values for across-patient validation against ILAE classification for subclinical and focal seizures. Outstanding performance is marked in bold. Unacceptable performance is marked in grey.
Shorthand: RP (model only uses random patient effects), RI (model includes both fixed marker and random patient effects using random intercept), RIS (model includes both fixed marker and random patient effects using random intercept and slope)

qualitatively agree with the results across all patients. Performance of markers across all patients and for TLE or eTLE patients does not differ greatly. It is expected that AUC values are slightly lower as the number of seizures considered is smaller when looking at each diagnosis in turn. These results suggest that markers can be equally applied for TLE and eTLE patients in an across-patient context. It is likely that the difference in markers resulting from different seizure onset zones is captured in the random patient effects.

**Marker**	RP	RI	RIS
Line length	NaN	**0.917**	**0.974**
Energy	NaN	**0.952**	**0.977**
δ band-power	NaN	**0.951**	**0.970**
θ band-power	NaN	**0.963**	0.866
α band-power	NaN	**0.949**	0.862
β band-power	NaN	**0.934**	**0.976**
Low-γ band-power	NaN	0.890	**0.970**
High-γ band-power	NaN	**0.950**	**0.966**
Prop. chan. included	NaN	**0.971**	**0.971**
Prop. chan. at MR	NaN	**0.971**	**0.972**
Time to MR	NaN	**0.970**	**0.937**
Prop. of seizure to MR	NaN	**0.969**	**0.935**
Major. suppr. duration	NaN	0.798	0.715
Part. suppr. duration	NaN	**0.930**	0.850
Suppr. strength	NaN	**0.959**	0.771
Duration	NaN	**0.966**	**0.922**

Table S4.2: Table of area under the curve (AUC) values for across-patient validation against ILAE classification for focal aware and impaired awareness seizures. Outstanding performance is marked in bold.
Shorthand: RP (model only uses random patient effects), RI (model includes both fixed marker and random patient effects using random intercept), RIS (model includes both fixed marker and random patient effects using random intercept and slope)

S4.2 Within patients

Performance of markers in distinguishing focal *vs.* subclinical seizures was assessed using Wilcoxon rank sum tests for each patient. Effect sizes (r) were bound between zero and one, with values close to one suggesting strong effects between the seizure types. We next investigated potential confounding factors on effect sizes. Here we considered binary variables of patient sex, diagnosis (TLE *vs.* eTLE), and surgery outcome (good *vs.* bad), as well as continuous variables of disease duration, age, number of recording channels, and number of seizures recorded. For binary variables, Wilcoxon rank sum tests were used to assess effects between different patient groups. For continuous variables, we created linear regression models where the effect size is modelled by each variable ($r \sim$ variable). Table S4.6 displays r and p-values for all patient variables and each marker .

These results support that within-patient performance of markers is patient specific, with moderate to large effects between TLE and eTLE patients for six markers. Of the continuous variables, disease duration was found to impact all spatial markers. The number of recording channels had small effects for proportions of channels included and at maximum recruitment. The number of seizures recorded increased effect sizes for energy, δ, β, proportion of channels included, and proportion of channels at maximum recruitment. Age did not have any clear effects.

**Marker**	RP	RI	RIS
Line length	0.844	**0.928**	0.844
Energy	0.858	**0.943**	0.777
δ band-power	0.662	**0.962**	**0.943**
θ band-power	0.853	**0.943**	0.818
α band-power	0.800	**0.931**	**0.957**
α band-power	0.876	**0.928**	0.842
Low-γ	0.806	**0.943**	**0.981**
High γ	0.798	**0.971**	0.750
Prop. chan. included	NaN	**0.991**	**0.991**
Prop. chan. at MR	NaN	**0.992**	**0.992**
Time to MR	0.560	**0.977**	**0.983**
Prop. of seizure to MR	0.501	**0.977**	**0.984**
Major. suppr. duration	0.828	0.827	0.808
Part. suppr. duration	0.631	**0.981**	NaN
Suppr. strength	0.783	**0.913**	**0.992**
Duration	0.733	**0.972**	**0.981**

Table S4.3: Table of area under the curve (AUC) values for across-patient validation against ILAE classification for focal and FTBTC seizures. It should be noted that only six FTBTC seizures were recorded across all patients. Due to this small sample size, we can only interpret these results as indicative. Outstanding performance is marked in bold. Unacceptable performance is marked in grey.
Shorthand: RP (model only uses random patient effects), RI (model includes both fixed marker and random patient effects using random intercept), RIS (model includes both fixed marker and random patient effects using random intercept and slope)

**Marker**	RP	RI	RIS
Line length	0.819	0.772	0.618
Energy	0.768	0.609	0.638
δ band-power	0.795	0.617	0.650
θ band-power	0.688	0.785	0.500
α band-power	NaN	0.781	0.635
β band-power	0.778	0.747	0.644
Low-γ band-power	0.857	0.671	0.594
High-γ band-power	0.897	0.631	0.543
Prop. chan. included	**0.916**	0.523	0.523
Prop. chan. at MR	**0.917**	0.503	0.502
Time to MR	NaN	0.830	0.514
Prop. of seizure to MR	NaN	0.849	0.529
Major. suppr. duration	0.873	0.798	0.717
Part. suppr. duration	NaN	**0.958**	0.819
Suppr. strength	NaN	**0.973**	0.870
Duration	NaN	0.809	0.551

Table S4.4: Table of area under the curve (AUC) values for TLE-only across-patient validation against ILAE classification for subclinical and focal seizures. Outstanding performance is marked in bold. Unacceptable performance is marked in grey.
Shorthand: RP (model only uses random patient effects), RI (model includes both fixed marker and random patient effects using random intercept), RIS (model includes both fixed marker and random patient effects using random intercept and slope)

Results suggest that performance of markers is differently impacted by various patient features. This finding supports testing the library of markers on each patient to determine if their performance is adequate for the individual.

Repeating this analysis comparing focal seizures with and without impaired awareness. Fig. S4.2A shows a heat-map of r-values, Fig. S4.2B shows r-values with associated p-value less than 0.05. Only six patients met inclusion criteria for this analysis. In one patient (U15), there are large effects with $p < 0.05$ in at least three markers. For patients U22 and G12 two spatial markers (proportion of channels included and at maximum recruitment) had large effect sizes ($r > 0.8$, $p < 0.05$). Unlike our focal *vs.* subclinical analysis, there is not a clear distinction between TLE and eTLE patients. Further studies with a larger cohort are required to confirm these findings. It was not possible to test differences in effect sizes based on patient metadata as too few patients met inclusion criteria.

S4.3 Capturing fluctuations of seizure severity

In this paper, we used our markers to capture and assess changes in seizure severity on circadian and longer timescales. Circular-linear correlation was used to assess changes of severity across the day, Table S4.7 presents circular-linear correlation values for peak markers, all other markers are

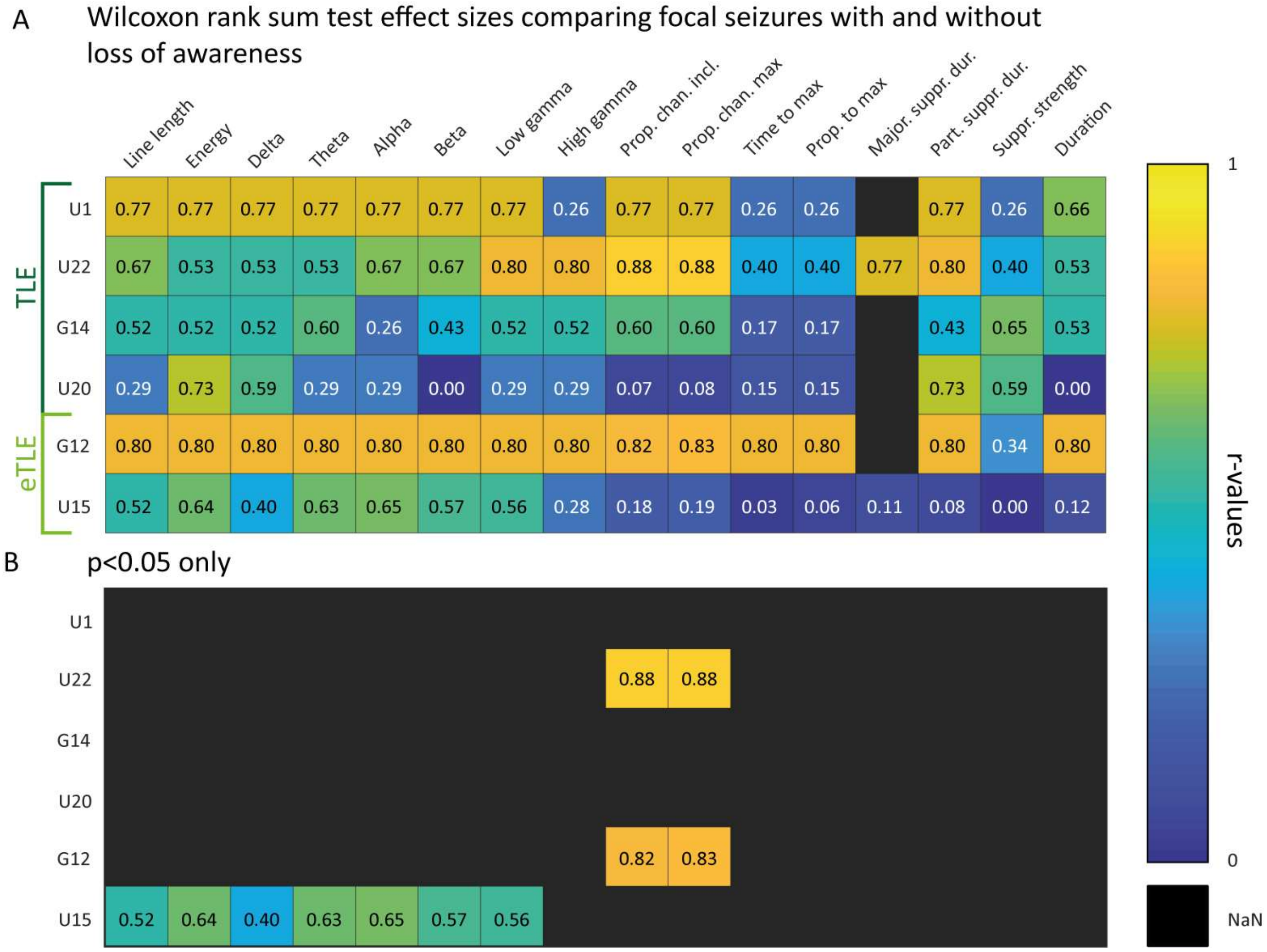

Figure S4.2: Validating markers against focal seizures with and without impaired awareness on a within-patient basis. A) Heat-map of Wilcoxon Rank Sum r values comparing focal seizures with and without impaired awareness within patients. B) Heat-map of Wilcoxon Rank Sum r values comparing focal seizures with and without impaired awareness within patients with only r-values with associated p-value < 0.05.

Marker	RP	RI	RIS
Line length	0.567	0.873	0.888
Energy	0.547	0.871	0.899
δ band-power	0.623	0.873	0.896
θ band-power	0.538	0.887	0.885
α band-power	0.551	0.859	0.883
β band-power	0.557	0.889	**0.906**
Low-γ band-power	0.569	0.872	NaN
High-γ band-power	0.593	0.871	NaN
Prop. chan. included	0.656	0.870	0.888
Prop. chan. at MR	0.644	0.871	0.884
Time to MR	0.643	**0.904**	**0.911**
Prop. of seizure to MR	0.605	0.886	0.895
Major. suppr. duration	0.630	0.879	0.881
Part. suppr. duration	0.501	0.876	0.886
Suppr. strength	0.510	0.861	0.877
Duration	0.734	**0.924**	**0.947**

Table S4.5: Table of area under the curve (AUC) values for eTLE-only across-patient validation against ILAE classification for subclinical and focal seizures. Outstanding performance is marked in bold. Unacceptable performance is marked in grey.
Shorthand: RP (model only uses random patient effects), RI (model includes both fixed marker and random patient effects using random intercept), RIS (model includes both fixed marker and random patient effects using random intercept and slope)

shown in Table S4.8. P-values were calculated using permutation tests (1000 permutations). For each permutation, marker values were randomly reassigned, creating 'null' models. The proportion of the distribution of 'null' test statistics greater than the statistic obtained from the real data was the p-value. Correlations with $p < 0.05$ are shown in bold. Note this is reported for reference, and hence no FDR has been applied. For reference, the probability an individual having at least two or three correlations with $p < 0.05$ by chance given 1 marker (i.e., there was no correlation present) was approximately 18.9% or 4.3% respectively given $\alpha = 0.05$. Changes in seizure severity on longer timescales were captured using Spearman's rank correlation between the time of seizure occurrence with respect to the commencement of recording and the marker values. Tables S4.9 and S4.10 show correlation values for peak and all other markers, respectively.

**Marker**	r_{sex}	r_{TLE}	$r_{surgicaloutcome}$	$p_{dis.dur.}$	p_{age}	p_{nchan}	p_{nsz}
Line length	0.085	0.276	0.000	0.716	0.525	0.515	0.060
Energy	0.085	0.242	0.220	0.156	0.172	0.648	**0.042**
δ band-power	0.017	0.276	0.220	0.219	0.083	0.641	**0.018**
θ band-power	0.017	0.311	0.000	0.288	0.377	0.671	0.071
α band-power	0.222	0.069	0.110	0.662	0.321	0.581	0.097
β band-power	0.017	0.276	0.000	0.403	0.848	0.171	**0.020**
Low-γ band-power	0.222	0.345	0.220	0.947	0.968	0.911	0.150
High-γ band-power	0.085	0.207	0.275	0.476	0.257	0.989	0.135
Prop. chan. included	0.188	**0.552**	0.165	**0.002**	0.571	**0.034**	**0.009**
Prop. chan. at MR	0.256	**0.552**	0.055	**0.004**	0.435	0.064	**0.006**
Time to MR	0.120	**0.552**	0.165	**0.006**	0.541	0.246	0.285
Prop. of seizure to MR.	0.290	0.483	0.385	**0.002**	0.460	0.260	0.247
Major. suppr. duration	NaN	NaN	NaN	NaN	NaN	NaN	NaN
Part. suppr. duration	0.222	0.138	0.275	0.216	0.957	0.356	0.349
Suppr. strength	0.085	0.069	0.055	0.903	0.421	0.841	0.202
Duration	0.120	0.173	0.165	0.161	0.681	0.162	0.532

Table S4.6: Comparing Wilcoxon rank sum r values (i.e., effect sizes) across different patient groups. Wilcoxon rank sum was used to compare r values for categorical variables of sex, surgery outcome (ILAE 1 and 2 *vs.* ILAE 3+), and TLE *vs.* eTLE. Resultant r values are presented in first two columns. For continuous variables of disease duration, age, the number of recording channels, and the number of seizures recorded, linear regression with response variable r and continuous patient variables as response variable. Resultant p-values for explanatory variables are presented in columns three to six. Effect sizes with $p > 0.05$, and $p-$values > 0.05 are marked in bold.

**Patient ID**	Line length	Energy	δ band-power	θ band-power	α band-power	β band-power	Low γ band-power	High γ band-power
U9	**0.275**	0.048	0.027	**0.226**	**0.358**	**0.366**	**0.482**	**0.342**
U13	0.050	0.033	0.094	0.037	0.017	0.091	0.095	0.062
U14	0.070	**0.354**	**0.351**	0.075	0.018	**0.129**	0.026	0.068
U15	**0.262**	**0.253**	**0.161**	**0.145**	**0.245**	**0.320**	**0.284**	**0.171**
U19	0.011	0.010	0.007	0.001	0.015	0.079	**0.123**	0.071
U22	**0.375**	0.250	**0.325**	0.184	0.217	**0.338**	**0.509**	**0.310**
U26	0.035	**0.370**	0.234	0.204	0.008	0.040	**0.348**	0.133
U28	0.087	0.044	0.007	0.014	0.037	0.028	**0.159**	0.120
U36	**0.159**	0.021	0.045	**0.293**	**0.153**	0.021	**0.099**	0.040
U43	0.053	**0.370**	**0.237**	0.028	**0.251**	0.145	0.062	0.055
U46	0.023	0.033	0.052	0.036	0.002	0.050	0.009	0.015
U48	0.073	0.042	0.085	0.022	0.034	0.005	0.003	0.135
G4	**0.323**	**0.271**	**0.234**	**0.152**	**0.303**	**0.259**	**0.276**	0.034
G8	**0.172**	**0.079**	**0.207**	**0.152**	**0.087**	**0.113**	0.025	**0.068**
G11	**0.134**	0.003	0.004	0.020	0.010	**0.101**	**0.181**	0.016

Table S4.7: Circular linear correlation between markers and time of day of seizure occurrence for peak markers. Correlations with $p < 0.05$ based on permutation test with 1000 permutations marked in bold.

**Patient ID**	Prop. ch. incl.	Prop. ch. at MR	Time to MR	Prop. to MR	Major. suppr. dur.	Part. suppr. dur.	Suppr. strength	Duration
U9	**0.336**	**0.399**	0.009	0.012	0.125	0.146	0.191	0.019
U13	0.084	0.064	0.006	0.043	NaN	0.135	0.158	0.047
U14	0.023	0.027	**0.281**	**0.249**	0.692	0.009	0.057	**0.263**
U15	0.050	0.025	0.053	0.030	**0.423**	0.096	0.016	0.124
U19	0.073	0.040	0.014	0.019	0.006	0.051	0.097	0.005
U22	**0.404**	**0.423**	0.176	0.167	0.712	0.120	**0.304**	0.083
U26	0.171	0.139	0.080	0.078	NaN	0.057	0.142	0.007
U28	0.047	0.039	0.026	0.021	NaN	0.030	0.010	0.126
U36	0.004	0.013	0.038	0.012	0.118	0.064	0.014	**0.292**
U43	0.127	0.101	0.041	0.045	0.019	0.060	0.008	0.077
U46	0.073	0.131	0.039	0.122	NaN	0.072	0.124	0.091
U48	0.017	0.094	0.164	0.151	**0.406**	**0.373**	**0.220**	0.135
G4	0.033	0.052	0.038	0.054	0.034	**0.246**	**0.210**	0.002
G8	0.012	0.041	**0.228**	**0.203**	**0.313**	**0.153**	0.006	**0.258**
G11	0.014	0.013	**0.075**	**0.064**	0.159	0.003	0.031	**0.074**

Table S4.8: Circular linear correlation between markers and time of day of seizure occurrence for 'spatial' and suppression markers, and duration. Correlations with $p < 0.05$ based on permutation test with 1000 permutations marked in bold.

**Patient ID**	Line length	Energy	δ band-power	θ band-power	α band-power	β band-power	Low γ band-power	High γ band-power
U9	0.320	0.223	0.084	0.303	0.212	**0.458**	0.359	**0.444**
U13	0.123	0.310	**0.487**	0.211	0.095	0.048	0.015	0.004
U14	**0.296**	**0.424**	**0.426**	**0.271**	0.086	0.099	0.226	0.078
U15	**0.499**	**0.653**	**0.581**	**0.611**	**0.576**	**0.590**	**0.565**	**0.289**
U19	**0.273**	**0.763**	**0.759**	**0.550**	**0.524**	0.178	0.204	**0.479**
U22	0.278	**0.507**	**0.671**	0.229	0.102	0.281	0.367	0.011
U26	**0.442**	**0.457**	**0.438**	**0.483**	0.408	**0.509**	**0.457**	**0.479**
U28	0.073	**0.497**	0.279	0.015	0.049	0.012	**0.320**	0.096
U36	0.121	**0.510**	**0.471**	**0.572**	**0.404**	**0.366**	0.037	0.093
U43	0.139	0.291	0.287	0.182	0.221	0.077	0.158	**0.371**
U46	0.240	**0.757**	**0.760**	**0.474**	0.152	0.006	0.263	**0.466**
U48	0.211	0.025	0.327	0.037	0.283	0.357	0.254	0.122
G4	0.116	0.023	0.056	0.079	0.055	0.151	**0.336**	0.165
G8	0.086	0.018	0.000	0.017	0.103	0.058	0.072	0.058
G11	0.001	0.159	**0.236**	**0.228**	0.060	0.152	0.118	**0.236**

Table S4.9: Spearman's rank correlation ρ between peak markers and time in EMU for patients with ≥ 20 recorded seizures. Correlations with $p < 0.05$ marked in bold

**Patient ID**	Prop. ch. incl.	Prop. ch. at MR	Time to MR	Prop. to MR	Major. suppr. dur.	Part. suppr. dur.	Suppr. strength	Duration
U9	**0.574**	**0.611**	0.191	0.193	NaN	0.230	0.207	0.080
U13	**0.416**	**0.407**	0.167	0.173	NaN	0.091	0.201	0.169
U14	0.165	0.132	0.161	0.148	NaN	0.015	0.032	0.213
U15	0.049	0.011	0.084	0.041	NaN	0.195	0.028	0.235
U19	0.008	**0.383**	0.239	**0.324**	**0.326**	0.066	**0.503**	0.871
U22	0.356	0.407	0.257	0.239	NaN	0.050	0.294	0.196
U26	0.223	0.187	0.080	0.047	NaN	**0.533**	0.346	0.348
U28	0.049	0.060	0.137	0.155	NaN	0.072	0.145	0.078
U36	0.120	0.104	0.109	0.118	NaN	**0.250**	0.084	0.117
U43	0.187	0.133	0.056	0.023	NaN	0.176	0.024	0.291
U46	0.340	0.356	0.366	0.320	NaN	0.128	**0.429**	0.233
U48	0.238	**0.460**	0.133	0.339	0.203	0.067	0.081	**0.374**
G4	**0.264**	0.229	**0.415**	**0.379**	NaN	0.057	0.096	**0.402**
G8	0.060	0.059	**0.279**	**0.268**	NaN	0.066	0.149	**0.249**
G11	**0.287**	**0.279**	**0.249**	**0.243**	NaN	**0.261**	0.095	0.173

Table S4.10: Absolute Spearman's rank correlation ρ between spatial, suppression & duration markers, and time in EMU for patients with ≥ 20 recorded seizures. Correlations with $p < 0.05$ marked in bold
